# An AI-based automatic leukemia classification system utilizing dimensional Archimedes optimization

**DOI:** 10.1038/s41598-025-98400-6

**Published:** 2025-05-16

**Authors:** Warda M. Shaban

**Affiliations:** 1 Department Communication and Electronics Engineering, Nile Higher Institute for Engineering and Technology, Mansoura, Egypt; 2 Faculty of Artificial Intelligence and Informatics, Horus University, New Damietta, Egypt

**Keywords:** Leukemia, Classification, Machine learning, Feature selection, Data mining, Data processing, Cancer

## Abstract

Leukemia is a common type of blood cancer marked by the abnormal and uncontrolled proliferation and expansion of white blood cells. This anomaly impacts the blood and bone marrow, diminishing the bone marrow’s capacity to generate platelets and red blood cells. Abnormal red blood cells in the bloodstream harm various organs, such as the kidneys, liver, and spleen. Detection and classification of infected patients at an early stage can save their lives. In this paper, a new Artificial Intelligence (AI) system is proposed. The proposed system is called Leukemia Classification System (LCS). The proposed LCS composed of five stages, which are; (i) Image Processing Stage (IPS), (ii) Image Segmentation Stage (ISS), (iii) Feature Extraction Stage (FES), (iv) Feature Selection Stage (FSS), and (v) Classification Stage (CS). During IPS, the input images are preprocessed through several processes: resizing, enhancement, and filtering. Next, the preprocessed images are segmented through ISS. Then, two types of features, texture and morphological features, are extracted. We feed these extracted features to FSS, which uses a proposed method to select the most important and effective features. The proposed method is called the Dimensional Archimedes Optimization Algorithm (DAOA). DAOA is based on the Archimedes Optimization Algorithm (AOA) and Dimensional Learning Strategy (DLS). Actually, DLS transmits valuable information about the ideal position of the population in every generation to the personal best position of each individual particle. This improves both the precision and efficiency of convergence while reducing the likelihood of the “two steps forward, one step back” phenomenon. This problem offers a more precise solution. Finally, these selected features are fed to the proposed classification model. Experimental results show that the proposed LCS outperforms the others.

## Introduction

**Fig. 1:**
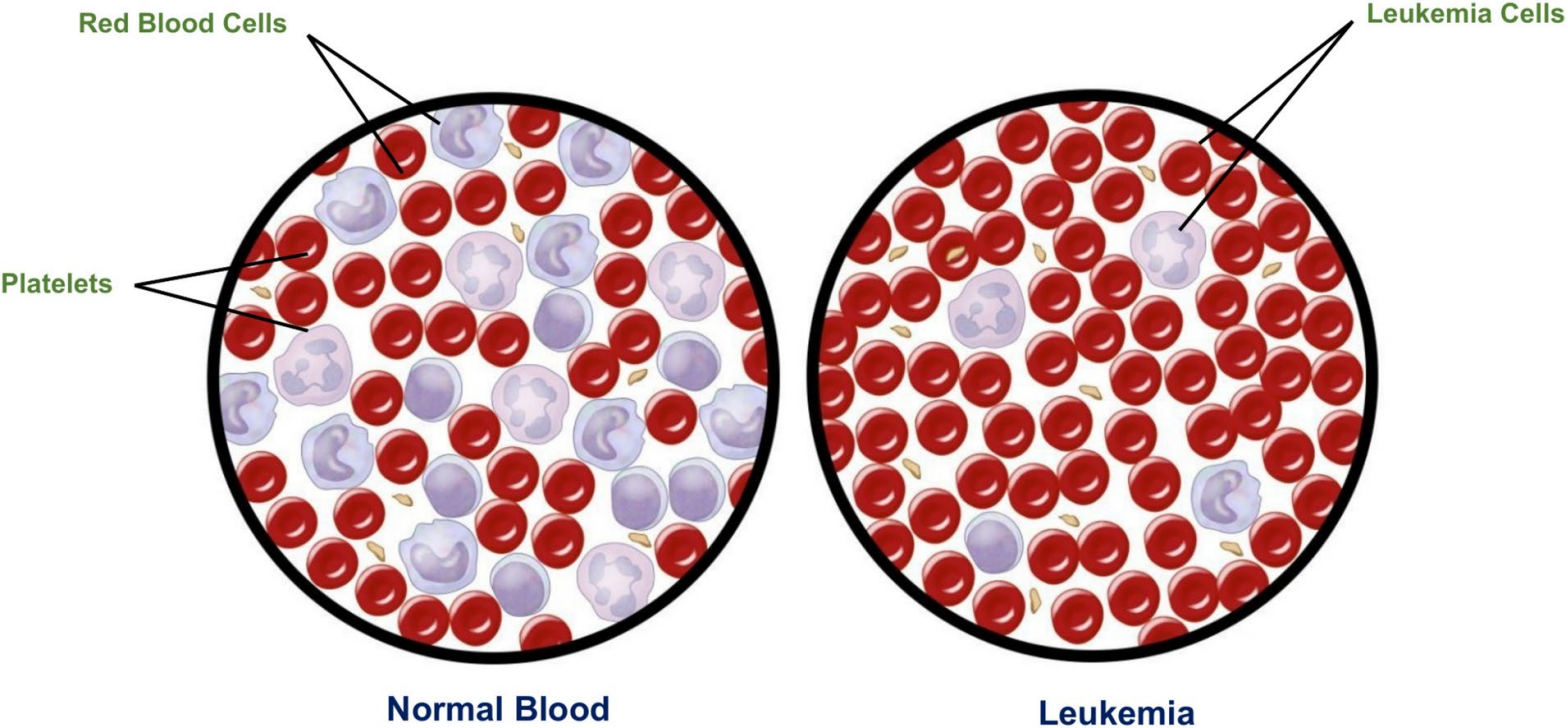
Shows the difference between normal blood and leukemia^2^.

Leukemia is a type of cancer that originates from cells that would normally develop into different kinds of blood cells. Leukemia typically originates from White Blood Cells (WBCs) as shown in Fig. 1, although certain types of leukemia can also originate from other blood cell types^1^. The main classification of leukemia depends on its acuteness (rapid growth) or chronicity (slower growth), as well as whether it originated in myeloid or lymphoid cells. Accurate identification of the particular form of leukemia enables physicians to more accurately forecast an individual’s prognosis and determine the most optimal course of treatment^2^.

**Fig. 2:**
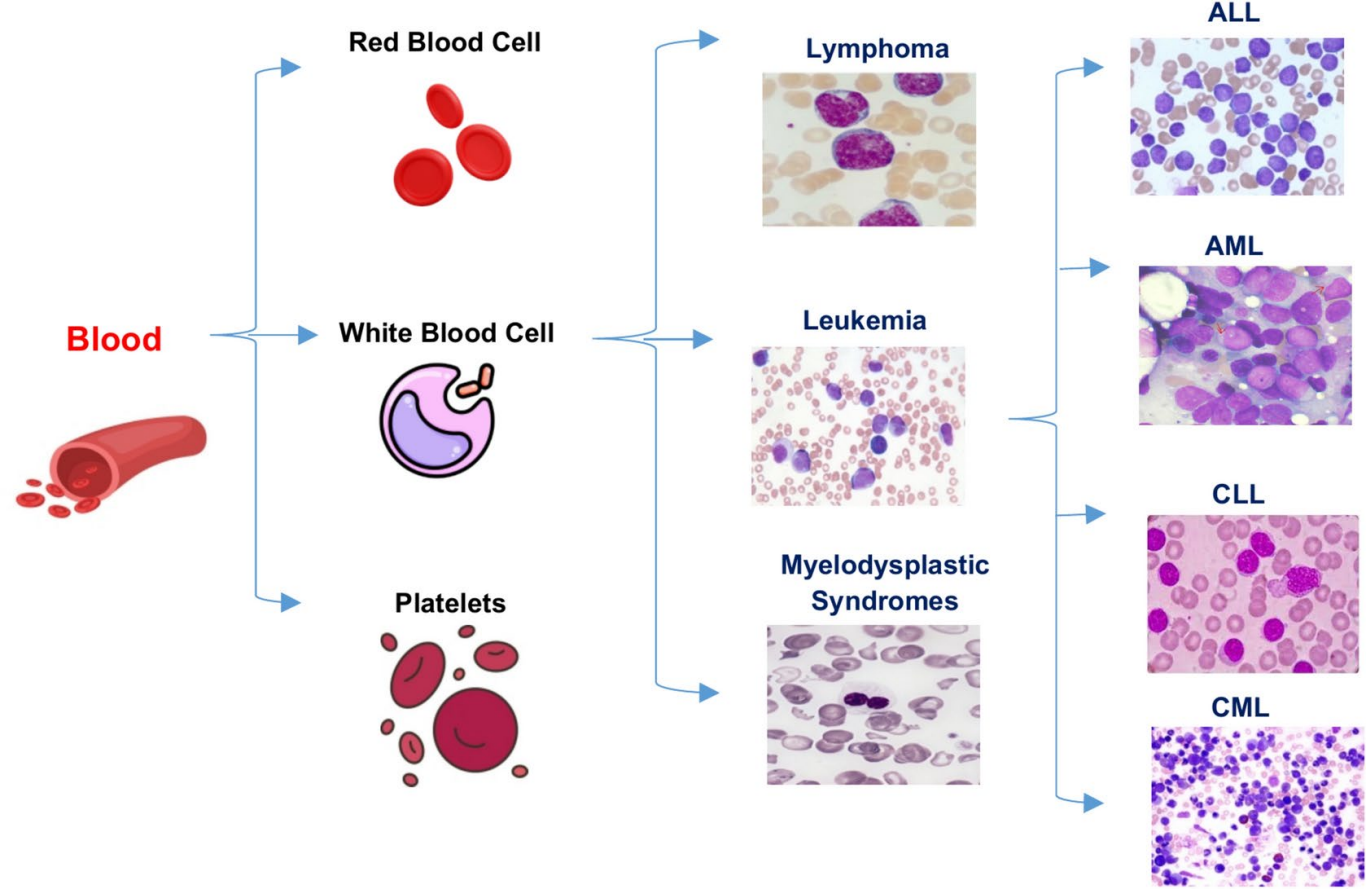
Shows the classification of blood and leukemia^3^.

Figure 2 shows that leukemia encompasses a wide range of distinct types. Certain conditions are prevalent among children, whereas others are more prevalent among adults. The choice of treatment is contingent upon the specific classification of leukemia and additional variables. Actually, leukemia is classified by healthcare providers based on the rate at which the disease progresses and the specific type of blood cell that is affected. As shown in Fig. 2, leukemia can be classified into four primary categories^3^:

- Acute Lymphocytic Leukemia (ALL) is the predominant form of leukemia among individuals aged 0 to 39, encompassing children, teenagers, and young adults. ALL can impact individuals of all age groups, including adults.
- Acute Myelogenous Leukemia (AML) is the prevailing form of acute leukemia among adults. Prevalence is higher among individuals aged 65 and above. AML can also manifest in pediatric patients.
- Chronic Lymphocytic Leukemia (CLL) is the predominant form of chronic leukemia among adults, particularly those aged 65 and above. The manifestation of symptoms in CLL may be delayed for several years.
- Chronic Myelogenous Leukemia (CML) primarily affects older adults, particularly those over the age of 65, although it can also occur in adults of any age. It is infrequent in children. The onset of symptoms in CML may be delayed for several years.

Researchers, doctors, and hematologists have long faced the challenge of diagnosing leukemia at an early stage. The symptoms of leukemia include lymph node enlargement, pallor, fever, and weight loss. However, it is important to note that these symptoms can also be present in other diseases^4^. Diagnosing leukemia in its early stages is challenging due to the subtle manifestation of symptoms. The most prevalent method for diagnosing leukemia is through the microscopic examination of peripheral blood smears (PBS). However, the gold standard for leukemia diagnosis entails the collection and analysis of bone marrow samples.

Moreover, the process of diagnosing and classifying leukemia in this manner incurs significant expenses and requires a substantial amount of time. Furthermore, the precision of diagnosis and categorization is contingent upon the expertise and proficiency of specialists in this domain. Machine Learning (ML) methods in computer vision can effectively address these challenges and problems^5^. By employing ML techniques, it is possible to automatically diagnose and classify acute leukemia with greater ease and at a reduced cost^6^.

ML is a prominent area of artificial intelligence that utilizes algorithms and mathematical relationships. It has been quickly embraced in the realm of clinical research. ML enables computers to learn from experience rather than being explicitly programmed^7^. The user did not provide any text. The utilization of these techniques in medical data processing has yielded exceptional results, leading to significant achievements in disease diagnosis^8,9^. Studies show that in the field of medical image processing, ML techniques significantly enhance intricate medical decision-making processes by extracting and subsequently analyzing the characteristics of these images^8,10^. With the proliferation of medical diagnosis tools and the generation of a substantial amount of high-quality data, there has been a pressing demand for more sophisticated data analysis techniques. Conventional techniques were incapable of analyzing a vast amount of data or identifying data patterns^11^.

In this paper, a new diagnosis system based on ML is introduced to classify leukemia patients. The proposed system is called Leukemia Classification System (LCS). Actually, LCS consists of four main stages, which are; (i) Image Processing Stage (IPS), (ii) Segmentation Stage (SS), (iii) Feature Extraction Stage (FES), and (iv) Classification Stage (CS). In the first stage (i.e., IMS), the used image is preprocessed through several processes, which are; image resizing, image enhancement, filtering and image smoothing. Next, the preprocessed image is segmented to in order to isolate WBCs from other cellular components such as red blood cells and particles in the background. Then, the features are extracted from the segmented image. Actually, two types of features are used; morphological features and texture features. These features are fused together and fed to the next stage (i.e., FSS). In FSS, the most informative and effective features are selected form those extracted features. Through FSS, a new feature selection method is proposed called Dimensional Archimedes Optimization Algorithm (DAOA). DAOA is a method that merges the Dimensional Learning Strategy (DLS) with the Archimedes Optimization Algorithm (AOA). AOA is a physics-based optimization technique that utilizes meta-heuristics to solve real-world problems by optimizing quantifiable factors like performance, profit, and quality. The AOA offers the benefits of reduced parameters, a straightforward interface, and straightforward implementation. However, there are also certain limitations, such as a deficiency in the variety of search and exploration abilities, and a tendency to become trapped in local optima. Consequently, DLS is used to mitigate these issues. DLS allows every aspect of a particle’s individual optimal solution is influenced by the equivalent aspect of the best solution found by the entire population. Then, these selected features are fed to the proposed classification model through CS. In CS, Ensemble Classifiers (EC) are used to classify leukemia patients based on their maximum voting. The experimental results showed that the suggested LCS performed better than the alternative strategies.

The paper is structured as follows: Section “Problem definition” presents the problem definition. Section “Preliminary concepts” covers the principles outlined in this article. Section “Related work” gives a summary of the prior research. Section “The proposed leukemia classification system (LCS)” demonstrates the proposed Leukemia Classification System (LCS). Section “Experimental results” presents the results acquired. Section “Discussion” presents the discussion of the obtained results. Section “Conclusions and future works” will cover conclusions and future works.

## Problem definition

In recent decades, researchers have focused on the topic of Blood Peripheral (PB) images and have put forth numerous ML algorithms. Actually, multiple endeavors have been undertaken to develop machine learning systems that aid in the diagnosis and classification of ALL^12–14^. The shared characteristic among the published approaches is that their diagnostic effectiveness relies on the extraction of a relatively high number of features in order to carry out a precise training process and classification for the systems. This factor enhances the likelihood of noise manifestation as a result of excessively fitting the data points, leading to an escalation in the expended effort and time of the classification process. Nevertheless, there is a scarcity of studies in the literature that incorporate algorithms relying on a limited number of features.

The current study aims to present a quick and straightforward classification system for automatically distinguishing between different ALL subtypes. At first, the used dataset images are analyzed using an adaptive segmentation algorithm to identify the primary characteristics. The extracted features are utilized to train and test robust classifiers by considering all possible permutation cases. This procedure allows us to identify the minimum set of features that result in the highest diagnostic accuracy. Various evaluation metrics are used to assess the effectiveness of automatic classification based solely on selected features.

## Preliminary concepts

The principles used in this article include; Archimedes optimization algorithm (AOA), and Dimensional Learning Strategy (DLS) which are covered in detail in the following subsections.

### Archimedes optimization algorithm (AOA)

**Fig. 3:**
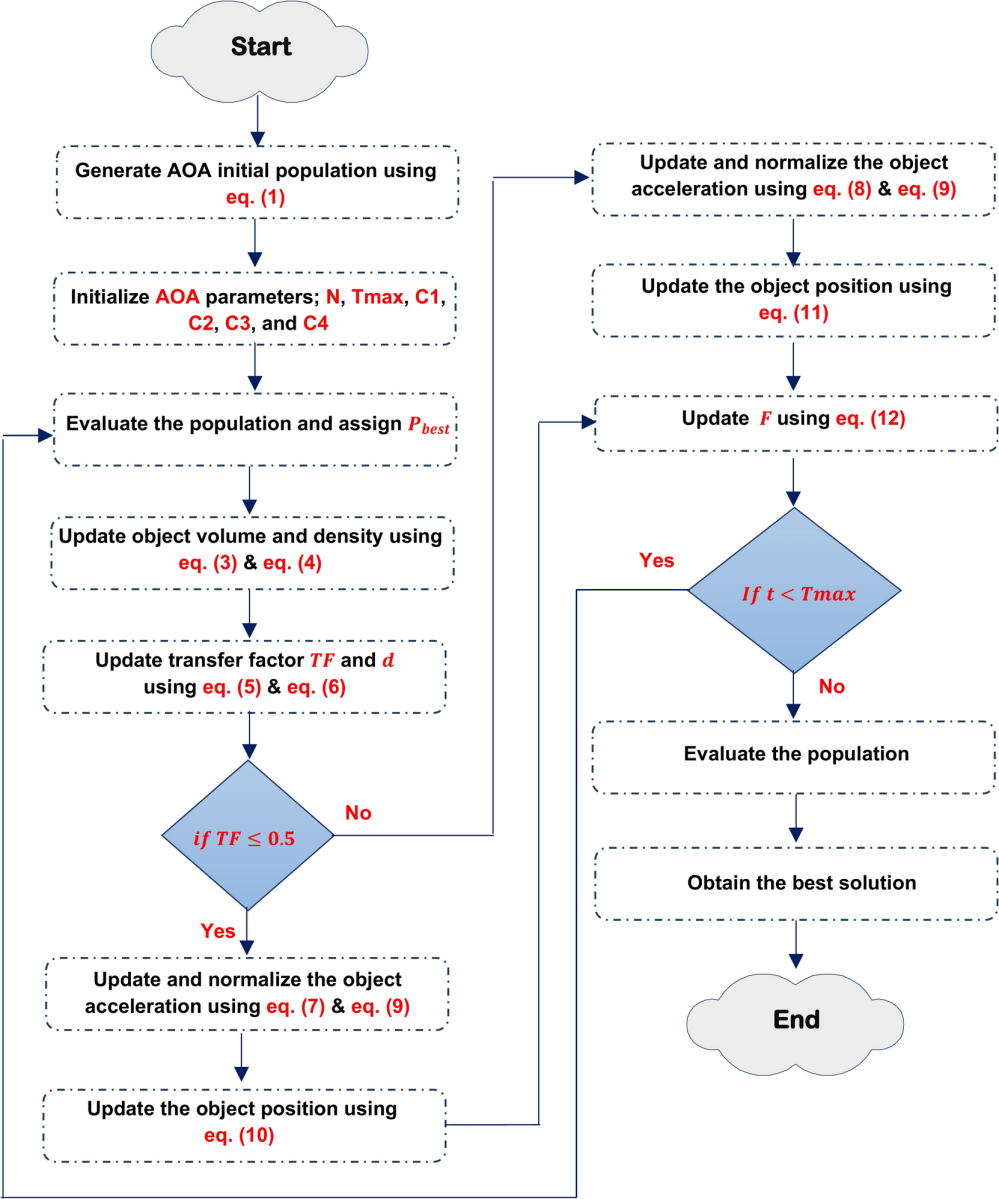
Shows the flowchart of conventional AOA.

Archimedes Optimization Algorithm (AOA) is a physics-inspired algorithm that draws inspiration from Archimedes’ law. The method was developed by Fatma Hashim in 2020 and falls under the category of metaheuristics^15^. The uniqueness of this technique resides in the encoding of the solution, which incorporates three auditory attributes: Volume $(V)$, Density $(D)$, and acceleration $(acc)$ for the fundamental agents. Therefore, the group of agents is originally produced in $D$ dimensions via a random process. Random values of $V$, $D$, and acc are presented as additive data. Following the evaluation procedure, each object is assessed to discover the most optimal one. Figure 3, shows the flowchart of AOA.

As shown in Fig. 3, the algorithm starts with generate initial population of $N$- object randomly using the following equation.

1 $$Ob_{i} = Ob_{{i_{Min} }} + rand \left( {Ob_{{i_{Max} }} - Ob_{{i_{Min} }} } \right),\quad i = 1,2, \ldots , N$$

where ${Ob}_{i}$ donates the ${i}^{th}$ object, ${Ob}_{{i}_{Max}}$ and ${Ob}_{{i}_{Min}}$ are the maximum and minimum limits of the search space, at the same order. Furthermore, each object ${Ob}_{i}$ is distinguished by its density ${D}_{i}$, Volume ${V}_{i}$, which are generated randomly and acceleration ${acc}_{i}$ which can be determined using Eq. (2):

2 $$acc_{i} = acc_{{i_{max} }} + r_{4} *\left( {acc_{{i_{max} }} - acc_{{i_{min} }} } \right)$$

Then, evaluate the initial population and determine the object with the maximum fitness value. Then, specify the ${Ob}_{best}$, ${V}_{best}$, ${D}_{best}$, and ${acc}_{best}$. Then update the volume and density for the next iteration using the following equations.

3 $$D_{i} \left( {t + 1} \right) = D_{i} \left( t \right) + rand*\left( {D_{best} - D_{i} \left( t \right)} \right)$$

4 $$V_{i} \left( {t + 1} \right) = V_{i} \left( t \right) + rand*\left( {V_{best} - V_{i} \left( t \right)} \right)$$

where ${D}_{i}\left(t+1\right)$, and ${D}_{i}\left(t\right)$ is the density of the *i*th object at try $(t+1)$, and try $(t)$ respectively. Additionally, ${V}_{i}\left(t+1\right)$, and ${V}_{i}\left(t\right)$ is the volume of the *i*th object at try $(t+1)$, and try $(t)$ respectively. Rand is a random value between 0 and 1. Operator of Transfer Factor $(TF)$ is employed to shift the focus of the search from exploration to exploitation, which can be calculated using the following equation.

5 $$TF = \exp \left( {\frac{{t - T_{\max } }}{{T_{\max } }}} \right)$$

where $T_{\max }$ denotes the highest possible number of iterations, whereas $t$ represents the current iteration number. In AOA, the TF gradually increases throughout each iteration until it reaches a value of 1. Similarly, the factor $df$, which reduces density, aids AOA in doing global-to-local searches which can be determined using the following equation.

6 $$d\left( {t + 1} \right) = \exp \left( {\frac{{t - T_{\max } }}{{T_{\max } }}} \right) - \left( {\frac{1}{{T_{\max } }}} \right)$$

where $d\left(t+1\right)$ represents the density of the $(t + 1$) iteration. The density diminishes gradually, allowing for convergence to a particular point. The density parameter is essential for achieving a balance between exploitation and exploration in AOA. The object’s acceleration is then adjusted based on Eqs. (7) and (8)^15^.

7 $$acc_{i} \left( {t + 1} \right) = \frac{{D_{r} + V_{r} *acc_{r} }}{{D_{i} \left( {t + 1} \right)*V_{i} \left( {t + 1} \right)}}\quad for\;TF \le 0.5$$

8 $$acc_{i} \left( {t + 1} \right) = \frac{{D_{best} + V_{best} *acc_{best} }}{{D_{i} \left( {t + 1} \right)*V_{i} \left( {t + 1} \right)}}\quad for\;TF > 0.5$$

where ${acc}_{i}\left(t+1\right)$ is object acceleration at the next try $(t+1)$, ${acc}_{best}$ represents the object beat acceleration. Additionally, ${D}_{i}\left(t+1\right)$, and ${V}_{i}(t+1)$ density, volume, and *i*th object. Moreover, ${D}_{r}, {V}_{r}$, and ${acc}_{r}$ are the acceleration, density, and volume of random material. Then the normalized acceleration can be calculated using the following equation:

9 $$acc_{i - norm} \left( {t + 1} \right) = u*\frac{{acc_{i} \left( {t + 1} \right) - \min \left( {acc} \right)}}{{\max \left( {acc} \right) - \min \left( {acc} \right)}} + 1$$

where $u$ and $l$ represent the normalization range, with values of 0.9 and 0.1, respectively. ${acc}_{i}\left(t+1\right)$ refers to incrementing the account number by 1. ${acc}_{i-norm}\left(t+1\right)$ is used to determine the proportion of change in each agent’s step. Finally, the position of each object is updated using Eq. (10)^15^.

10 $$P_{i} \left( {t + 1} \right) = P_{i} \left( t \right) + C_{1} *rand*acc_{i - norm} \left( {t + 1} \right)*d*\left( {P_{rand} - P_{i} \left( t \right)} \right)\quad for\;TF \le 0.5$$

11 $$P_{i} \left( {t + 1} \right) = P_{best} \left( t \right) + F*C_{2} *rand*acc_{i - norm} \left( {t + 1} \right)*d*\left( {T*P_{best} - P_{i} \left( t \right)} \right)\quad for\;TF > 0.5$$

where ${P}_{i}\left(t+1\right)$ is the object position at the next try $(t+1)$, and ${P}_{i}\left(t\right)$ is the position of *i*th object at $t$. Additionally, ${C}_{1}$, and ${C}_{2}$ are constant and their values equal to 2 and 6, respectively. T increases over time in direct proportion to the transfer operator, defined as $T={C}_{3}*TF$. The flag $F$ is used to change the direction of motion and can be determined by the following equation:

12 $$F = \left\{ \begin{gathered} + 1\quad if\;R \le 0.5 \hfill \\ - 1\quad if\;R > 0.5 \hfill \\ \end{gathered} \right.$$

where $R=2*rand-{C}_{4}$. Algorithm 1 illustrate the conventional AOA.

### Dimensional learning strategy (DLS)

Xu et al. introduced the Dimensional Learning Strategy (DLS) in order to protect the important information of particles in the Particle Swarm Optimization (PSO) algorithm^16^. The DLS architecture creates a learning exemplar for every particle. By learning from the corresponding part of the population’s optimal solution, each part of a particle’s optimal solution learns something new throughout the construction process. So, the learning exemplar does a great job of integrating the outstanding information gleaned from both individual and collective best practices. Take into consideration that ${P}_{best}$ represents the optimal object position at iteration $t$, ${P}_{global}$ denotes the optimal position in the entire population, and $Pdl$ denotes the output learning position. Algorithm 2 illustrates DLS.

**Algorithm 1:**
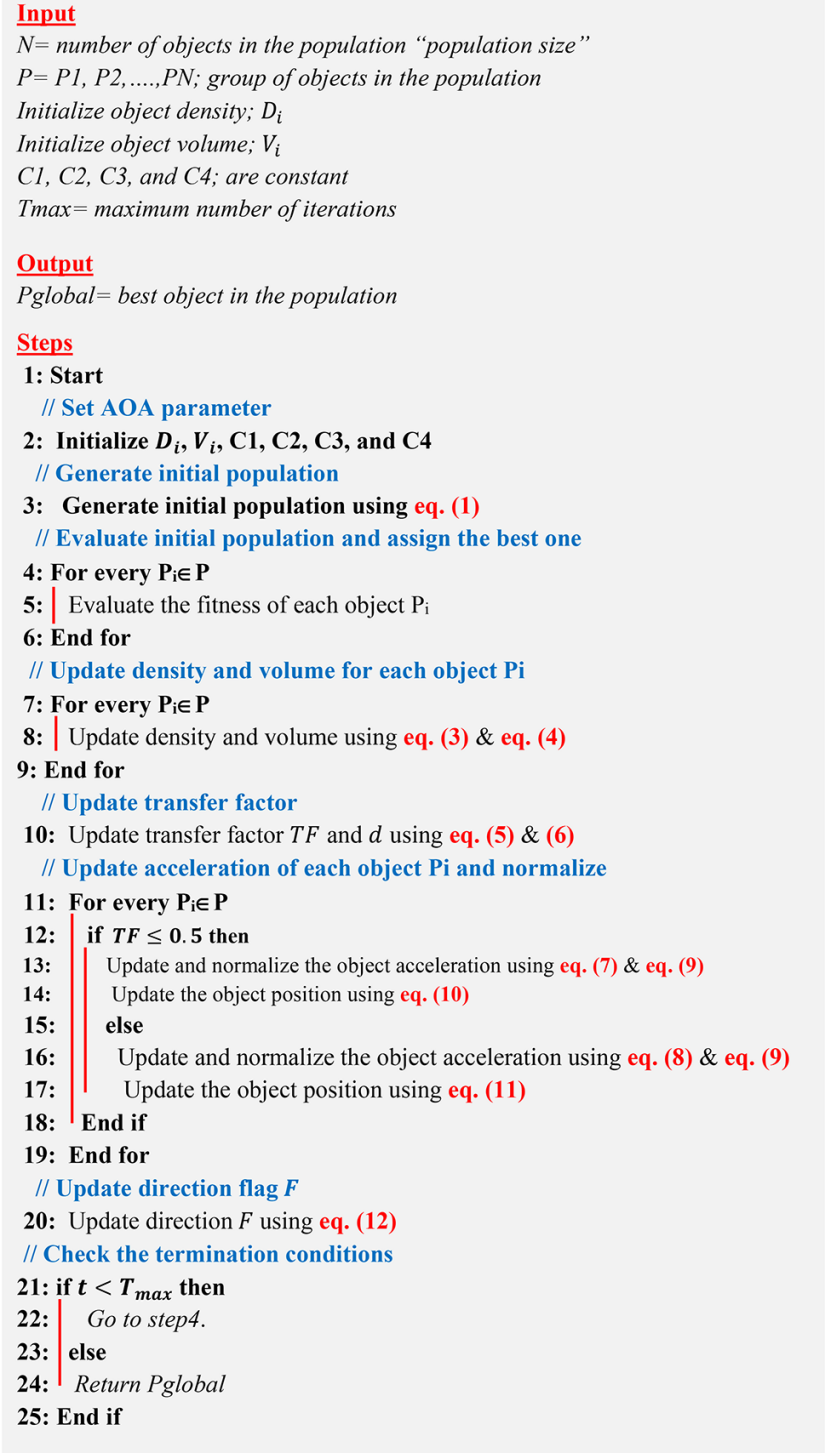
Conventional AOA

**Algorithm 2:**
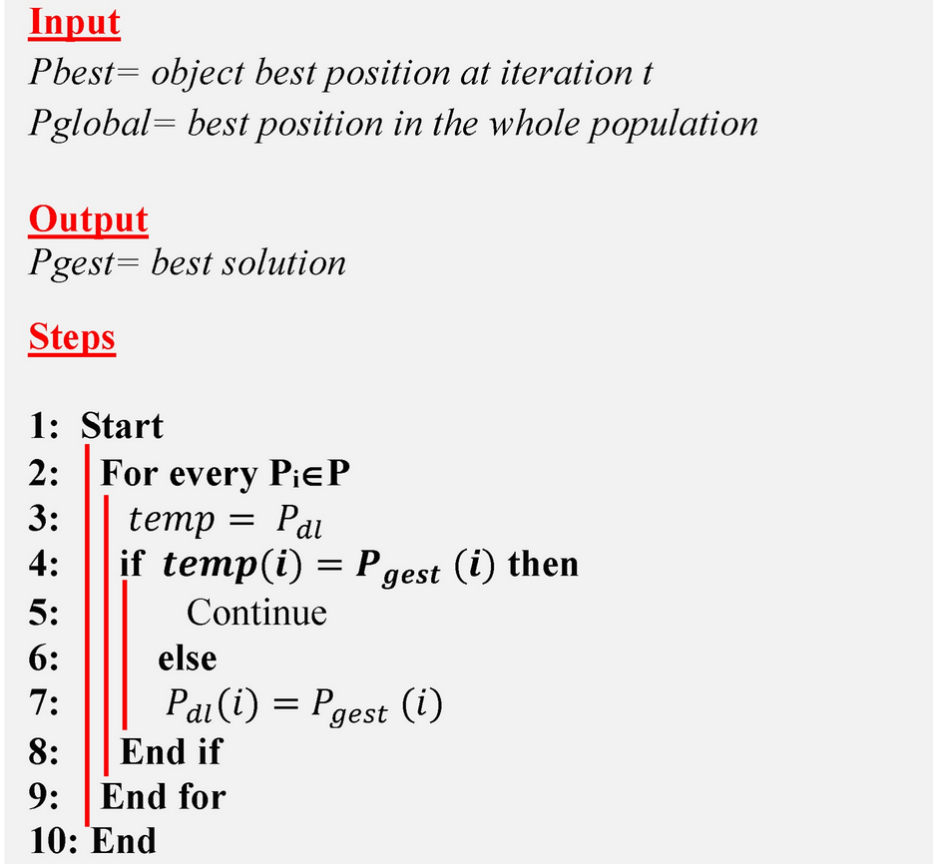
Dimensional Learning Strategy (DLS)

## Related work

This section will provide a detailed overview of the prior research on leukemia classification strategies. In order to properly categorize ALL from microscopic pictures of WBCs, a new Hybrid Model (HM) combining the Inception v3 and XGBoost algorithms was introduced in^1^. For feature extraction, the suggested model makes use of Inception v3, and for classification, it makes use of the XGBoost model. Based on the results of the empirical tests, the proposed model was superior to the other methods described in the literature. The suggested HM’s weighted F1 score is 0.986. The obtained results show that different CNNs’ classification accuracy is improved when an XGBoost classification head was used instead of a softmax head.

The study in^17^ introduces a non-intrusive method that employs Convolutional Neural Networks (CNNs) and medical images to carry out the diagnostic process. The suggested approach involves employing a CNN-based model that incorporates an attention module known as Efficient Channel Attention (ECA) in conjunction with the visual geometry group from Oxford (VGG16). This combination aims to extract high-quality deep features from the image dataset, resulting in improved feature representation and more accurate classification outcomes. The proposed method demonstrates that the ECA module effectively addresses the morphological similarities between images of ALL cancer cells and healthy cells. Diverse augmentation techniques are additionally utilized to enhance the caliber and volume of training data. The experimental findings demonstrate that the proposed CNN architecture effectively captures intricate characteristics and attains a precision level of 91.1%. The findings demonstrate that the suggested approach can be employed for diagnosing ALL and would assist pathologists.

Actually, Convolutional Neural Networks (CNNs) required a huge dataset consequently, the study in^18^ presented a highly effective deep CNNs framework to address this problem and achieve more precise ALL detection. Its improved speed and popularity as a preferred method are due to its key features, which include depth wise separable convolutions, linear bottleneck architecture, inverted residual, and skip connections. This proposed method introduces a new weight factor based on probability, which plays a crucial role in effectively combining MobilenetV2 and ResNet18 while retaining the advantages of both approaches. The performance is verified by utilizing publicly available benchmark datasets, namely ALLIDB1 and ALLIDB2. The experimental results demonstrate that the proposed approach achieves the highest level of accuracy, with 99.39% and 97.18% in the ALLIDB1 and ALLIDB2 datasets, respectively.

As presented in^19^, a novel model called ALNett, which utilizes a deep neural network and depth-wise convolution with varying dilation rates to accurately classify microscopic images of WBCs was proposed. The cluster layers consist of convolution and max-pooling operations, which are then followed by a normalization process. This process enhances the structural and contextual information in order to extract strong local and global features from the microscopic images. These features are crucial for accurately predicting ALL. The model’s performance was evaluated by comparing it to several pre-trained models, namely VGG16, ResNet-50, GoogleNet, and AlexNet. The experimental findings demonstrated that the ALNett model, as proposed, achieved the highest classification accuracy of 91.13% and an F1 score of 0.96, while also exhibiting reduced computational complexity.

Additionally, as introduced in^20^, DeepLeukNet**,** a CNN based microscopy adaptation model for ALL classification, was developed. The problem of overfitting in the model has been tackled by employing data augmentation techniques to generate more images. Subsequently, a qualitative analysis was conducted by visually examining the activation of the intermediate layer, ConvNet filters, and heatmap layers. A comparative study was conducted to validate the efficacy of our proposed model in comparison to existing methods. The experimental results demonstrated that the suggested model surpasses the previous works documented in the same field.

A classification method has been introduced in^21^ to understand the convergence of training Deep Neural Networks (DNN). The researchers utilized DNN to categorize the gene expression data. The study used a dataset containing the gene expressions of bone marrow from 72 individuals diagnosed with leukemia. A five-layer classifier was created to categorize samples of ALL and AML. 80% of the data was used for training the network, and the remaining 20% was set aside for validation. The DNN classifier is performing satisfactorily when compared to other classifiers. Two types of leukemia can be classified with 98.2% precision, 96.59% sensitivity, and 97.9% specificity.

As shown in^22^, an Innovative Framework Model (IFM) for segmenting and classifying leukemia using a Deep Learning (DL) structure has been introduced. The proposed system consists of two primary components: a DL technology for segmenting and extracting characteristics, and a classification system for analyzing the segmented section. A novel UNET architecture has been developed to facilitate segmentation and feature extraction procedures. Several performance factors, including precision, recall, F-score, and Dice Similarity Coefficient (DSC), were used to test the model on four different datasets. The segmentation and categorization tasks were performed with an impressive average accuracy of 97.82%. Furthermore, an impressive F-score of 98.64% was attained. The results obtained demonstrate that the method presented is a robust technique for detecting leukemia and classifying it into appropriate categories. In addition, the model surpasses certain implemented methods. Table 1 summarizes the pros and cons of the proposed leukemia classification methods from the aforementioned literature.

**Table 1:** A brief comparison of the current leukemia classification methods.

References	Description	Pros	Cons
Ramaneswaran et al.^1^	A new Hybrid Model (HM) was proposed for ALL classification. HM combines between Inception v3 and XGBoost. Inception v3 was used for feature extraction process. While XGBoost was used for classification	The proposed model demonstrates a high degree of accuracy and reliability in detecting acute lymphoblastic leukemia cells	The used dataset is limited
Ullah et al.^17^	A diagnostic support system that utilizes a CNN and an ECA module was used to accurately classify images of cancerous and healthy cells. The VGG16 framework was utilized to extract features from the source images. The ECA module was added after each convolutional block to enhance the importance of the extracted features from VGG16	Incorporating the attention module into deep learning architectures can result in a substantial improvement in performance	The proposed model suffers from high false positive rate
Das and Meher^18^	An innovative deep CNN framework was presented to enhance the efficiency of detecting and classifying ALL. A new method was suggested that incorporates a probability-based weight factor to merge MobilenetV2 and ResNet18 efficiently, while still benefiting from the strengths of both techniques	The proposed framework overcome the problem of required huge dataset	The proposed model suffers from time complexity
Jawahar et al.^19^	A new model which is called ALNett was introduced. The custom CNN architecture utilizes a series of stacked convolution layers to acquire hierarchical features, resulting in precise classification. The inclusion of batch normalization between these stacked clusters improves the extracted features, leading to improved classification performance. The normalization process improves the weights and learning process, leading to a substantial reduction in features within the stacked hierarchical clusters. As a result, all microscopic images can be classified accurately and quickly	ALNett exhibited encouraging performance in categorizing ALL and surpassed the performance of the other pre-trained models	The system is very complex
Saeed et al.^20^	CNN-based adaptation model DeepLeukNet classifies ALL using microscopy images. Data augmentation has been used to generate additional images to reduce model overfitting. After visualizing intermediate layer, ConvNet filter, and heatmap layer activation, a qualitative analysis was performed. The proposed model was also compared to existing methods to verify its efficacy	The proposed model provides high accuracy	The proposed model suffers from time complexity
Mallick et al.^21^	A new training Deep Neural Networks (DNN) has been proposed. Actually, the proposed DNN aims to classify leukemia types. Due to the substantial size of the gene expression data, a neural network with seven layers is developed to accurately classify two distinct forms of leukemia. The classification accuracy is significantly superior in comparison to other types of classifiers. The user’s text is very short and does not provide any information	DL models possess the ability to process and analyze extensive quantities of data, subsequently extracting significant features without human intervention	The proposed DNN doesn’t give the optimum accuracy
Alzahrani et al.^22^	A new algorithm that can distinguish between and classify two distinct forms of leukemia: ALL and AML has been introduced. The three main parts of the algorithm are image preprocessing, segmentation, and classification. The segmentation feature is complete. Classification procedures are carried out with the help of a newly constructed UNET that utilizes a U-shaped architecture	The proposed system can enhance the capabilities of healthcare providers in delivering their services	One significant obstacle faced during this research was the absence of all types of leukemia in the datasets, which were limited to ALL and AML only. Therefore, these datasets pose various challenges. Only two categories have varying dimensions, necessitating input resizing

### Existing gaps and challenges

- *Dataset Limitations*: Lack of substantial, high-quality, well-annotated datasets is one of the main obstacles still present in leukemia classification. Many studies, including those utilizing MobilenetV2 and ResNet18^18^ depend on publicly available datasets such as ALLIDB1 and ALLIDB2. Diversity, quality, and the quantity of annotated images are among the aspects of these sets that sometimes suffer. Greater and more varied datasets would enable DL models to generalize more effectively and raise performance standards.
- *Interpretability of DL Models*: Although DL models especially CNNs can reach great accuracy they often serve as "black boxes," which makes it challenging to know how decisions are taken. In medical domains, where clinical acceptance and trust hinge on explainability, this deficiency in transparency poses a challenge. Although certain models, like DeepLeukNet^20^, attempt to address this by visualizing intermediate layers and activation heatmaps, there remains potential for further advancement in enhancing the interpretability and explainability of these models.
- *Generalization Across Different Population Groups*: Models developed using datasets that inadequately reflect the diverse populations present in actual clinical environments, such as ALNett^19^ and DeepLeukNet^20^, may lack the capacity to generalize across various populations, resulting in potential biases and diminished accuracy in clinical applications. Variations in cell shape and gene expression can be ascribed to diverse demographics, including age, ethnicity, and geographical location.
- *Computational Efficiency*: Particularly CNNs, DL models sometimes call for large computational resources. Although many deep models still struggle with speed and memory use, models like ALNett and Mobilenet V2 seek to lower complexity. Particularly in resource- constrained settings like rural hospitals or smaller clinics, the trade-off between model complexity and efficiency continues to be a major difficulty. In this sense, more developments in lightweight architectures, pruning, or model compression could be beneficial.
- *Model Robustness and Real-Time Diagnosis*: Despite achieving high accuracy in controlled environments, the robustness of these models in real-time clinical settings remains uncertain. Convolutional Neural Networks may encounter difficulties with noisy, low-quality, or heterogeneous imaging data from real-world situations, while exhibiting superior performance under controlled laboratory conditions with uniform image quality. This inconsistency in robustness may hinder the application of these models in real-world settings where image quality fluctuates significantly.

## The proposed leukemia classification system (LCS)

**Fig. 4:**
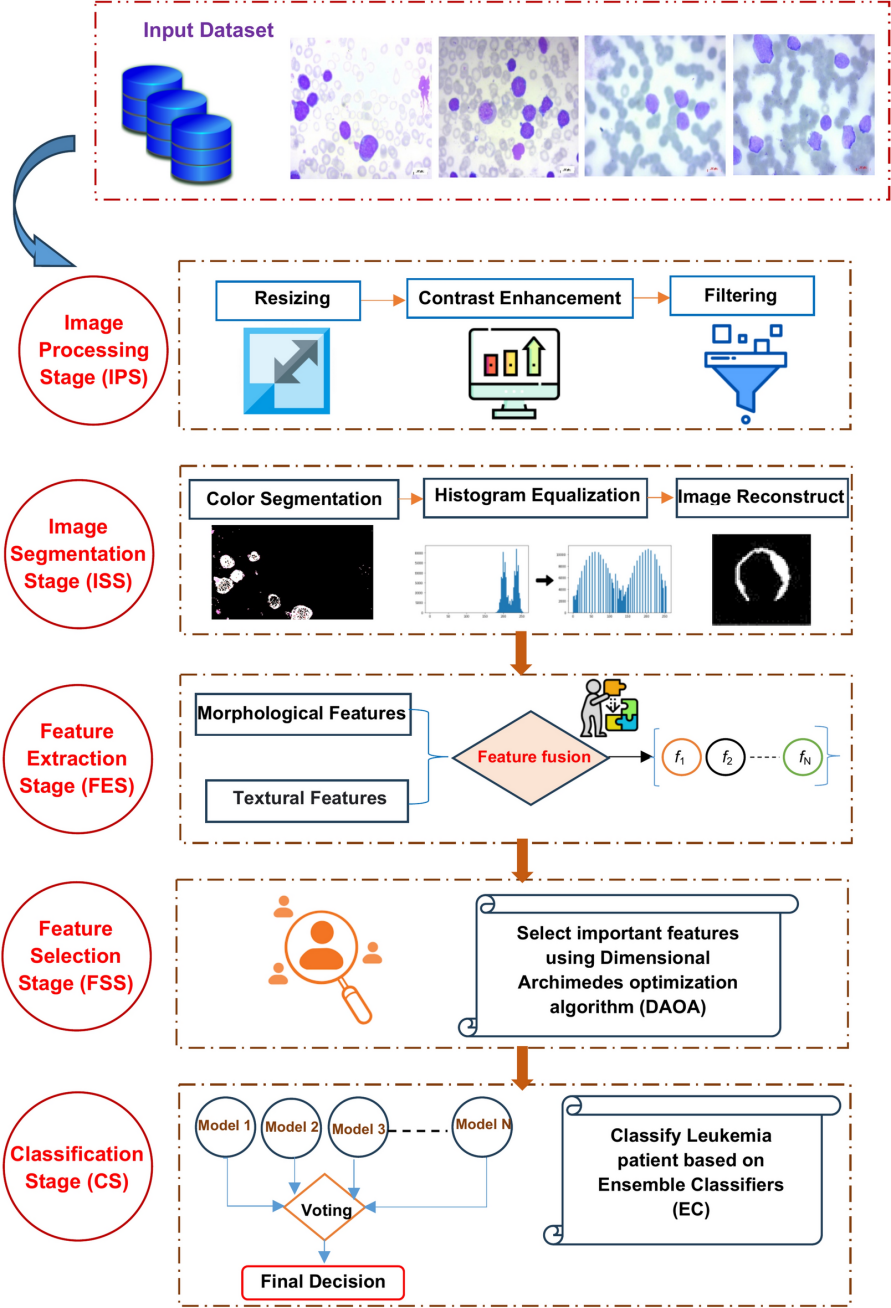
Shows the proposed LCS.

Leukemia mainly occurs in children and affects their tissues or plasma. Nevertheless, it has the potential to manifest in adults. If this disease is detected and diagnosed late, it can become lethal and result in death. Furthermore, leukemia can arise as a result of genetic mutations. Hence, early detection is imperative in order to preserve the patient’s life. In this paper, a new system was proposed to detect leukemia patients based on AI. As shown in Fig. 4, the proposed system is called Leukemia Classification System (LCS). Actually, LCS composed of four main stages, which are; (i) Image Processing Stage (IPS), (ii) Segmentation Stage (SS), (iii) Feature Extraction Stage (FES), and (iv) Classification Stage (CS). In the next subsection, each stage will be discussed in detail.

### Image processing stage (IPS)

In machine vision applications, preprocessing is a commonly employed procedure that has a direct impact on the efficacy of ML-based models. Preprocessing methods can assist in preparing the input data to reduce noise and enhance diagnosis^23^. In this paper, Image Preprocessing Stage (IPS) has been done via three processes which are, image resizing, image enhancement contrast, and filtering. The obtained images frequently had noise due to excessive irregularities caused by the staining process. The goal of IPS is to improve the image quality by removing unwanted objects and noise from the blood image prior to the segmentation process. IPS employed the following process^24^.

i. Resizing images: The RGB image was resized to 500 by 500 pixels to enhance computational efficiency.
ii. Image enhancement involved adjusting the image intensity to a normalized and scaled range of 0.5 to 0.9.
iii. Gaussian filter was used to reduce the effect of instrument noise (camera) and incorrect pixel values. This results in a uniform and evenly spread lighting pattern throughout the entire image.
iv. Image Smoothing: median and wiener filters were sequentially applied to the image to remove noise and enhance clarity.

### Image segmentation stage (ISS)

Image segmentation Stage (ISS) is a crucial area of study, particularly within the field of computer vision. Image segmentation in ML involves the partitioning of data into distinct groups. Because leukemia is characterized by aberrant WBCs, the most important step in the image analysis process is to separate the WBCs from other cell structures including, red blood cells and background particles. Precise findings in the following phases are the consequence of precise segmentation. The white blood cells, red blood cells and platelets were separated in our processing using the K-means algorithm^25^. The overlapping nucleated WBCs (lymphocytes and monocytes), on the other hand, were successfully separated using marker-controlled watershed segmentation algorithms that included erosion and dilation features. Because K-means runs quicker than Otsu and fuzzy C-means, it was chosen as the preferred approach. A portion of the leukocytes and monocytes’ cellular bodies were missing from the digital microscope photos, although some of them were visible around the sides of the images. A border cleaning method was used to exclude incomplete leukemia cell structures because it is challenging to extract features from them. Large inaccuracies in the picture analysis would be introduced if these partial monocytes and leukocytes were not deleted.

### Feature extraction stage (FES)

**Fig. 5:**
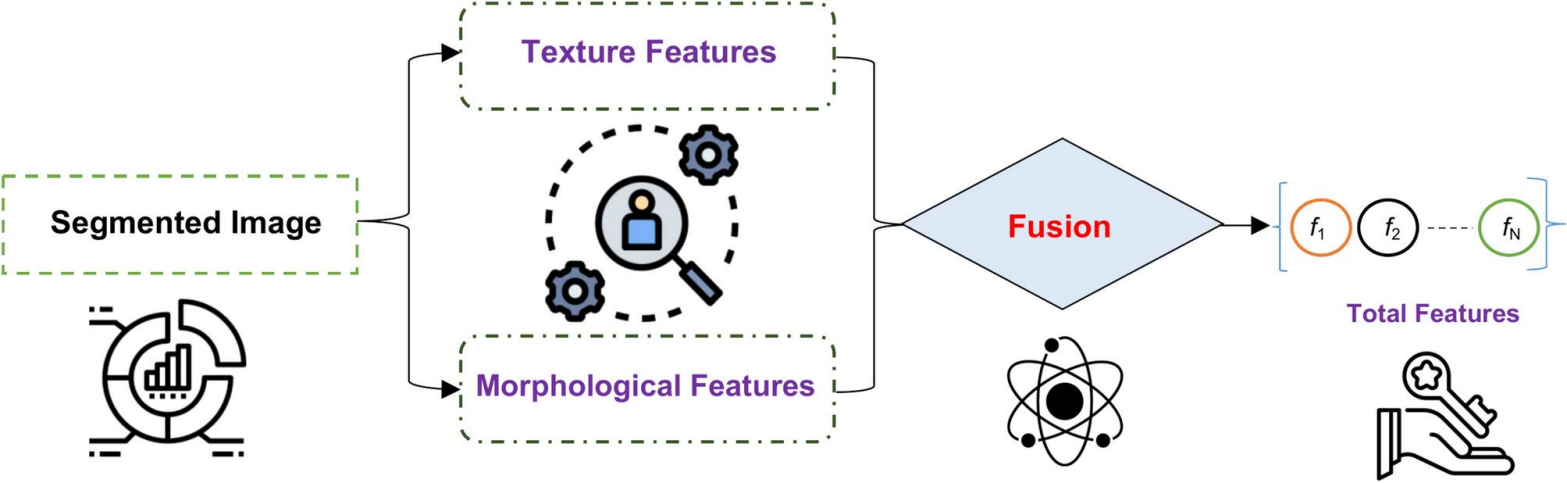
Shows the process of FES.

Feature Extraction Stage (FES) is the process of transforming the input image into a set of distinctive attributes. The main goal of FES is to decrease the number of features in a dataset by creating new features based on the existing ones and then removing the original features^26^. Indeed, the feature extraction process is carried out prior to utilizing the classification model. Utilizing features derived from the segmented image enhances the performance of the classification model, leading to accurate decision-making^26^. Currently, various methodologies are employed for the extraction of features. The techniques mentioned include Gabor filter, co-occurrence matrix, wavelet transform based-features, and others^26,27^. In this paper, FES was applied to the segmented image. In order to distinguish between normal and blast WBCs, feature extraction took into account cell size, count, color, shape, and chromatic structure the same criteria used by hematologists to distinguish between normal and blast white blood cells. Different features are extracted from the segmented image, such as texture features and morphological features. The process of FES is illustrated in Fig. 5.

#### Texture features

Texture features are the initial type of features extracted from the segmented image. This paper utilizes the Gray Level Cooccurrence Matrix (GLCM) for extracting texture features. GLCM is commonly used and very reliable for describing image texture in image analysis applications. Twenty-two GLCM features are extracted from various angles in this paper. The co-occurrence matrix is computed using a distance of d = 1 and applying the offset vector with angles of 0°, 45°, 90°, and 135°. The features include contrast, homogeneity, correlation, entropy, energy, cluster shade, cluster prominence, autocorrelation, maximum probability, sum of squares (variance), and others^10,28^.

#### Morphological features

Another kind of feature that is extracted from the segmented image are morphological features. Actually, extraction of morphological traits used by hematologists. The features encompass measurements of the area of the nucleus and cytoplasm, the perimeter of both the nucleus and the entire cell, the number of distinct parts of the nucleus, the average and variability of the boundaries of the nucleus and cytoplasm, and the ratio between the areas of the cytoplasm and nucleus. Furthermore, the roundness of the nucleus and the cell’s body is determined using the following formula^29^:

13 $$RC = \frac{{P_{e}^{2} }}{{4\Pi A_{r} }}$$

where ${P}_{e}$ is the perimeter and ${A}_{r}$ is the area of the region.

### Feature selection stage (FSS)

The key features are extracted through the Feature Selection Stage (FSS) after analyzing the segmented image. It is crucial to eliminate duplicate features from the extracted data once the feature extraction process is completed. Redundant features can complicate the training process, prolong it, and reduce the classifier’s performance^10,30^. Feature selection involves removing irrelevant, redundant, and unnecessary features to enhance the accuracy of the classifier being used. Therefore, FSS is crucial for enhancing the efficacy of learning algorithms. Indeed, feature selection is a crucial step in every medical diagnostic system. Feature selection methods are typically categorized into two main groups: filter and wrapper^28^. Filter methods are known for their speed and scalability, but they do not consistently deliver superior performance compared to wrapper methods. Wrapper methods offer superior performance but are more computationally expensive.

This paper introduces a novel feature selection technique named Dimensional Archimedes Optimization Algorithm (DAOA). DAOA is a method that merges the Dimensional Learning Strategy (DLS) with the Archimedes Optimization Algorithm (AOA). AOA is an innovative metaheuristic method that is based on the Archimedes principle, a basic law of physics. It was used to solve a meta-heuristic optimization problem with continuous constraints. AOA is transformed into Binary AOA (BAOA) to tackle the feature selection issue, categorized as a discrete optimization problem^29^. BAOA begins by establishing a group of solutions referred to as the Population (P). Each object represents a possible solution, such as the most efficient combination of features. The candidate solutions are represented as binary vectors containing values of “zero” or “one”. The magnitude of the vector is equivalent to the quantity of features. A value of “one” indicates selection, while a value of “zero” indicates deselection or removal in terms of features. An individual’s value determines the size of the optimal subset.

**Fig. 6:**
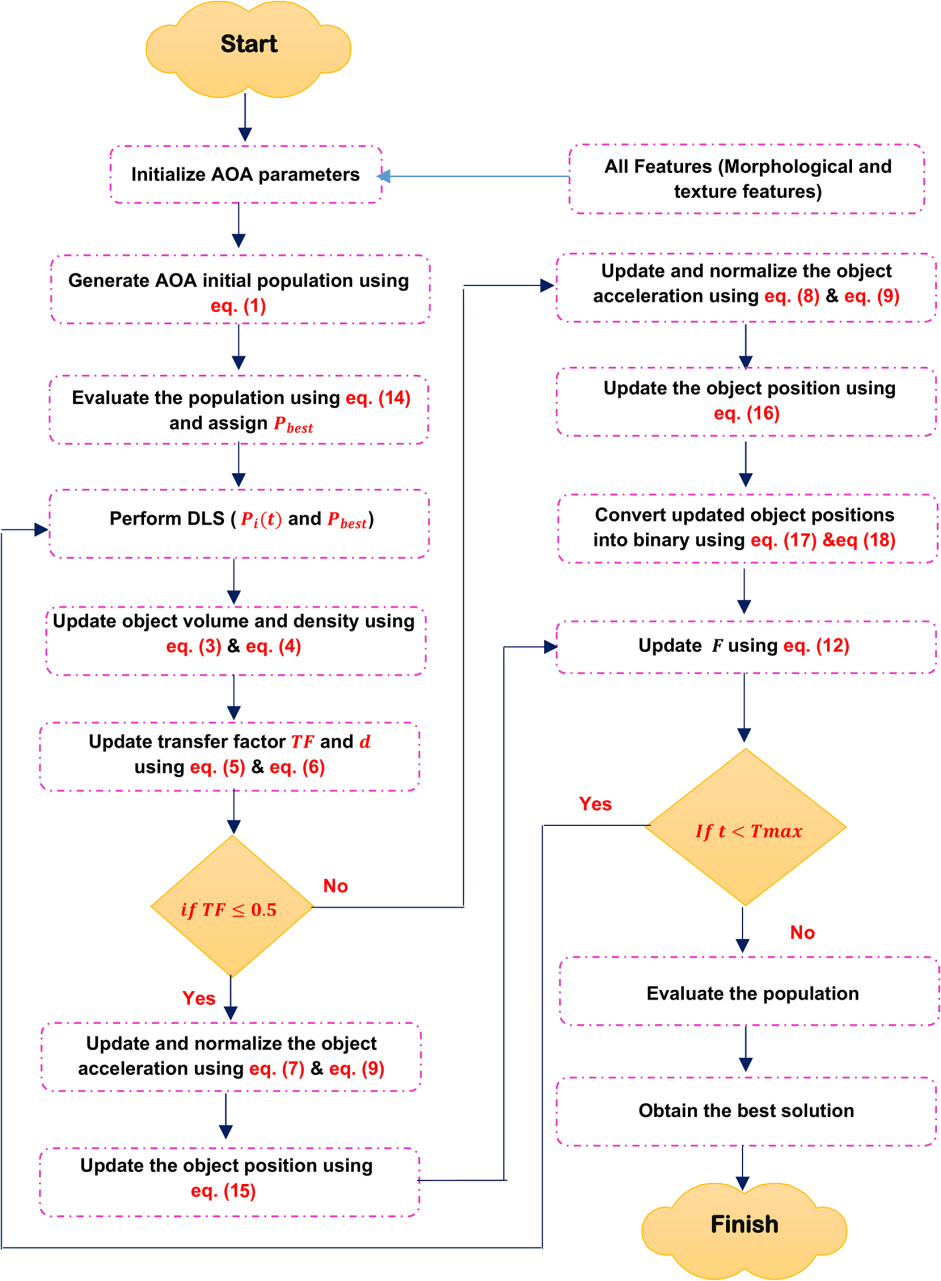
Shows the flowchart of the proposed DAOA.

Figure 6 shows that DAOA begins by extracting morphological and texture features from segmented images. The process then involves initializing the AOA parameters and generating the initial population of objects randomly using Eq. (1). Each object in the population represents a candidate solution. Next, evaluate the initial population using the fitness function. Fitness function is a way to evaluate the quality of the solution based on an accuracy of the used clasifier such as Naïve Bayes (NB) as a base classifier which can be calculated using the following equation:

14 $$F\left( {P_{i} } \right) = x*\left( {1 - \eta \left( {P_{i} } \right)} \right) + y*\left( \frac{M}{N} \right)$$

where $F\left({P}_{i}\right)$ is the fitness value of *i*th object and $\eta \left( {P_{i} } \right)$ is the classification accuracy using NB classifier. $M$ and $N$ It refers to the number of chosen features and the total number of features, respectively. $x$ and $y$ are constants. Next, after evaluating all objects, the best one is assigned as ${P}_{best}$ according to the maximum value.

**Fig. 7:**
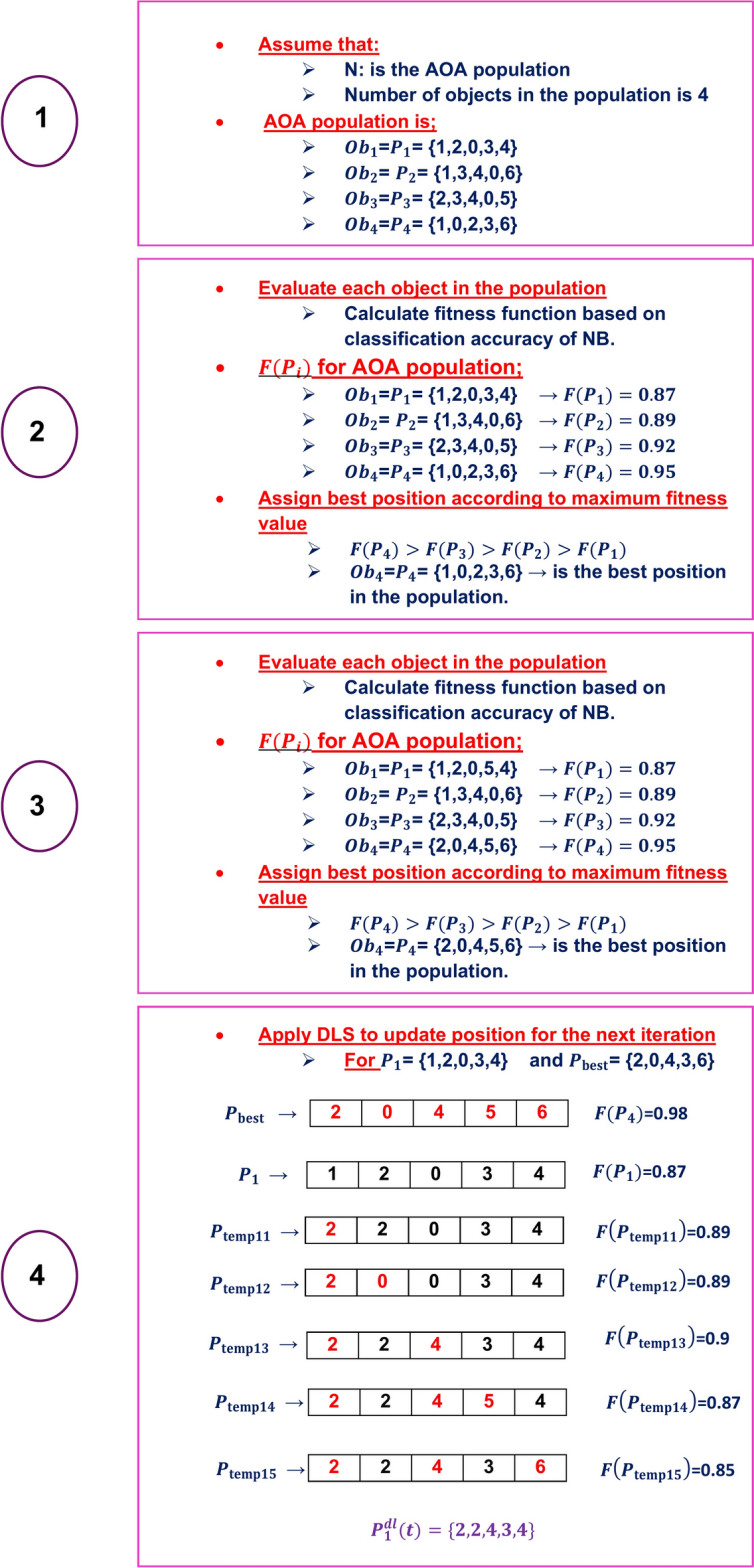
An illustrative example of DLS.

After identifying the optimal position, DLS is carried out. According to DLS with PSO^16^, the objects in the traditional AOA method gain knowledge from their personal best experience and the best experience of the whole population. This learning approach may lead to the occurrence of “oscillation” and "regression," characterized by periods of advancement followed by partial setbacks. Consequently, in this paper, DLS is applied to AOA to allow it to perform well and find the best solution. In DLS, the object learns from its position and the best position in the population. Once the best position in the population is determined, the conventional AOA procedure will proceed and update the object position using the following equations:

15 $$P_{i} \left( {t + 1} \right) = P_{i}^{dl} \left( t \right) + C_{1} *rand*acc_{i - norm} \left( {t + 1} \right)*d*\left( {P_{rand} - P_{i}^{dl} \left( t \right)} \right)\quad for\;TF \le 0.5$$

16 $$P_{i} \left( {t + 1} \right) = P_{best} \left( t \right) + F*C_{2} *rand*acc_{i - norm} \left( {t + 1} \right)*d*\left( {T*P_{best} - P_{i}^{dl} \left( t \right)} \right)\quad for\;TF > 0.5$$

where ${P}_{i}\left(t+1\right)$ is the position of ${i}^{th}$ object in the next try. ${P}_{i}^{dl}\left(t\right)$ is the position of the exemplar object. Figure 7 provides an illustrative example of how to apply DLS with AOA.

Then, after updating the object positions in the next try $(t+1)$, these positions are converted to binary to be suitable for feature selection problems. The binary position can be calculated using Eqs. (17) and (18)^31^.

17 $$Sig\left( {P_{i}^{j} \left( {t + 1} \right)} \right) = \frac{1}{{1 + e^{{ - \left( {P_{i}^{j} \left( {t + 1} \right)} \right)}} }}$$

18 $$P_{i}^{j} \left( {t + 1} \right) = \left\{ \begin{gathered} 0\quad if\;R \ge Sig\left( {P_{i}^{j} \left( {t + 1} \right)} \right) \hfill \\ 1\quad otherwise \hfill \\ \end{gathered} \right.$$

where ${P}_{i}^{j}\left(t+1\right)$ is the value of the *i*th object at the *j*th position in try $(t+1)$, where *j* = 1,2, 3,…,*m*, and rand (0,1) represents a random number between [0,1]. Moreover, the sigmoid transfer function $Sig\left( {P_{i}^{j} \left( {t + 1} \right)} \right)$ indicates the probability that the *j*th bit is either 0 or 1. After that, the procedure is carried over again for as many iterations as possible. The procedure ends after the population’s best object has been chosen. The most accurate indications of leukemia disease are all features in this item that have been supplied by one. The most important features that are selected using the proposed DAOA are GLCM features (contrast, homogeneity, correlation, entropy, energy, cluster shade, cluster prominence), morphological features (area, eccentricity, elongation, solidity, circularity, perimeter, and roundness). The proposed DAOA is shown in Algorithm 3.

**Algorithm 3:**
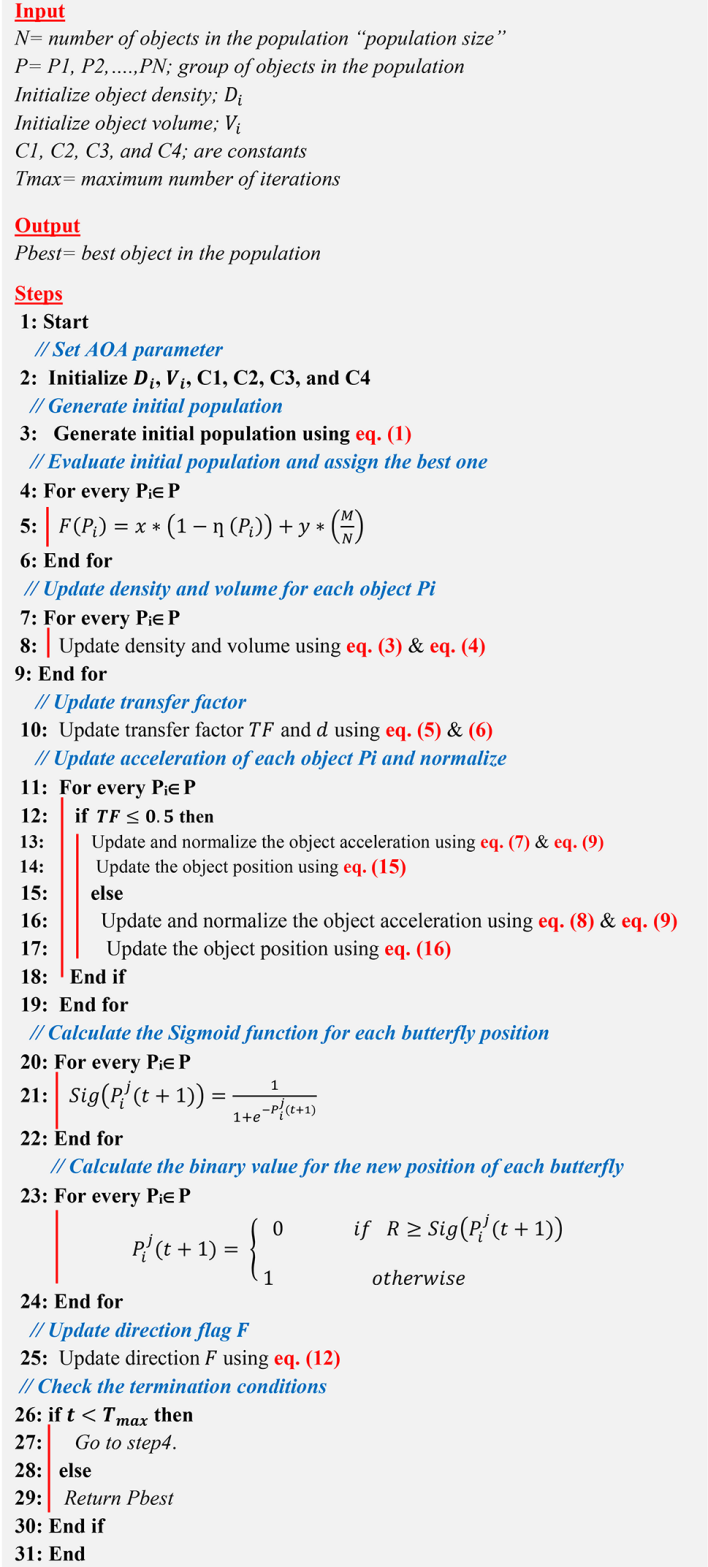
The proposed DAOA

### Classification stage (CS)

After identifying the most crucial features for classifying leukemia patients, ML is employed. ML technologies can be used to accurately and quickly identify leukemia patients at an early stage^32^. Using these technologies provides the advantage of producing immediate and precise results from automated detections. Time can be saved by utilizing the latest developments in computer vision and precision technology. This paper utilizes Ensemble Classifier (EC) to categorize leukemia patients employing various classifiers.

**Fig. 8:**
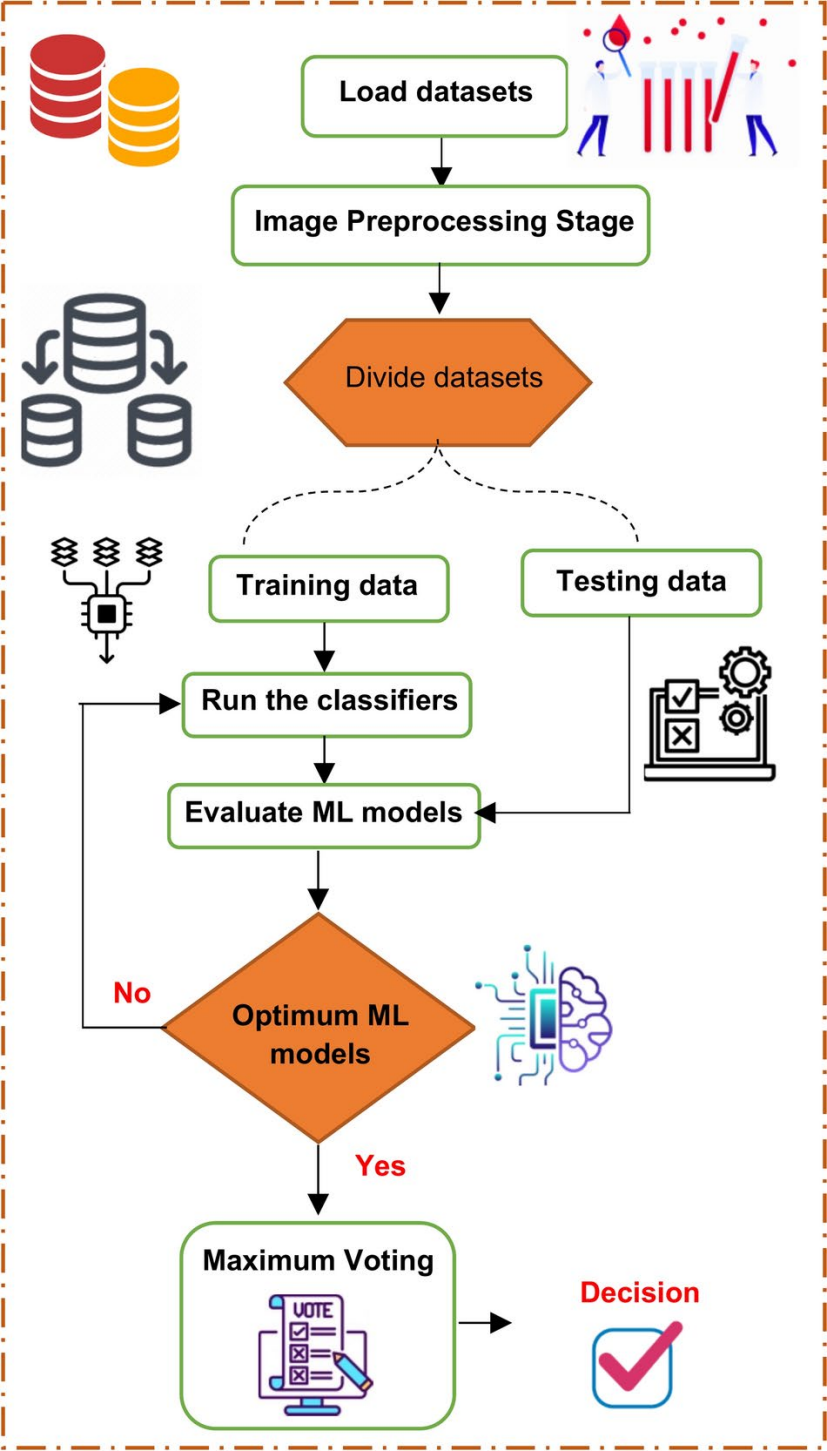
Shows the proposed classification model.

By combining numerous classifiers, EC aims to improve a weak classifier’s accuracy by decreasing its misclassification rate. Gathering the predictions of various classifiers from the initial data and combining them to form a robust classifier is the main concept. In this paper, the used classifiers are; Random Forest (RF), Decision Tree (DT), Gradient Boosting (GB), and Adaptive Boosting (AdaBoost) and the final decision is based on the maximum voting as shown in Fig. 8.

## Experimental results

In this part, we will evaluate the Leukemia Classification System (LCS) that has been proposed. The implementation of LCS involves five consecutive stages: Image Processing stage (IPS), Image Segmentation Stage (ISS), Feature Extraction Stage (FES), Feature Selection Stage (FSS), and Classification Stage (CS). Firstly, the used images are preprocessed by resizing, enhancing, and filtering the original image. Then, the region of interest is segmented through ISS. Then, within the context of FSS, the proposed Dimensional Archimedes Optimization Algorithm (DAOA) was introduced as a novel technique for identifying the most meaningful features. After that, the chosen features are fed to the proposed classification model through CS. In CS, an Ensemble Classifier (EC) is used to classify leukemia patients based on maximum voting. The implementation of the proposed system is based on a publicly available ALL data set^33,34^. The data has been used to make it easier to replicate the results that are reported in this article. There are two separate sets of data in the dataset: the training set and the testing set. To evaluate the proposed model the dataset was divided into 70% (2279 images) for training, and 30% (977 images) for testing. The parameters that were applied and the values that were implemented are shown in Table 2.

**Table 2:** The parameters applied with the corresponding used values.

Parameter	Description	Algorithm	Applied value
N	No. of features	–	34
$T_{\max }$	Maximum number of iterations		30
$x$	Constant	–	0.99
$y$			0.01
a	Control parameter	for GWO, SCA, and WOA	Linearly decrease from 2 to 0 over repetitions
r1, r2	Random vectors	for GWO	r1, r2 $\in$ [0,1]
c1, c2, c3	Random Values	for SSA	c1, c2, c3 $\in$ [0,1]
$\tau$ o	Initial Pheromone for each state for ACO	For ACO	0.2
ρ	Evaporation rate		0.2
α	The relative importance of the pheromone value		2
β	The relative importance of the heuristic information		1
${a}_{1}$	Constant	For HHO	${a}_{1}, {a}_{2}, {a}_{3}, {a}_{4}, p$ $\in$ [0,1]
$${a}_{2}$$			
$${a}_{3}$$			
$${a}_{4}$$			
$$p$$			
${C}_{1}$	Constant	For DAOA, AOA	2
${C}_{2}$			6

### Dataset description

**Fig. 9:**
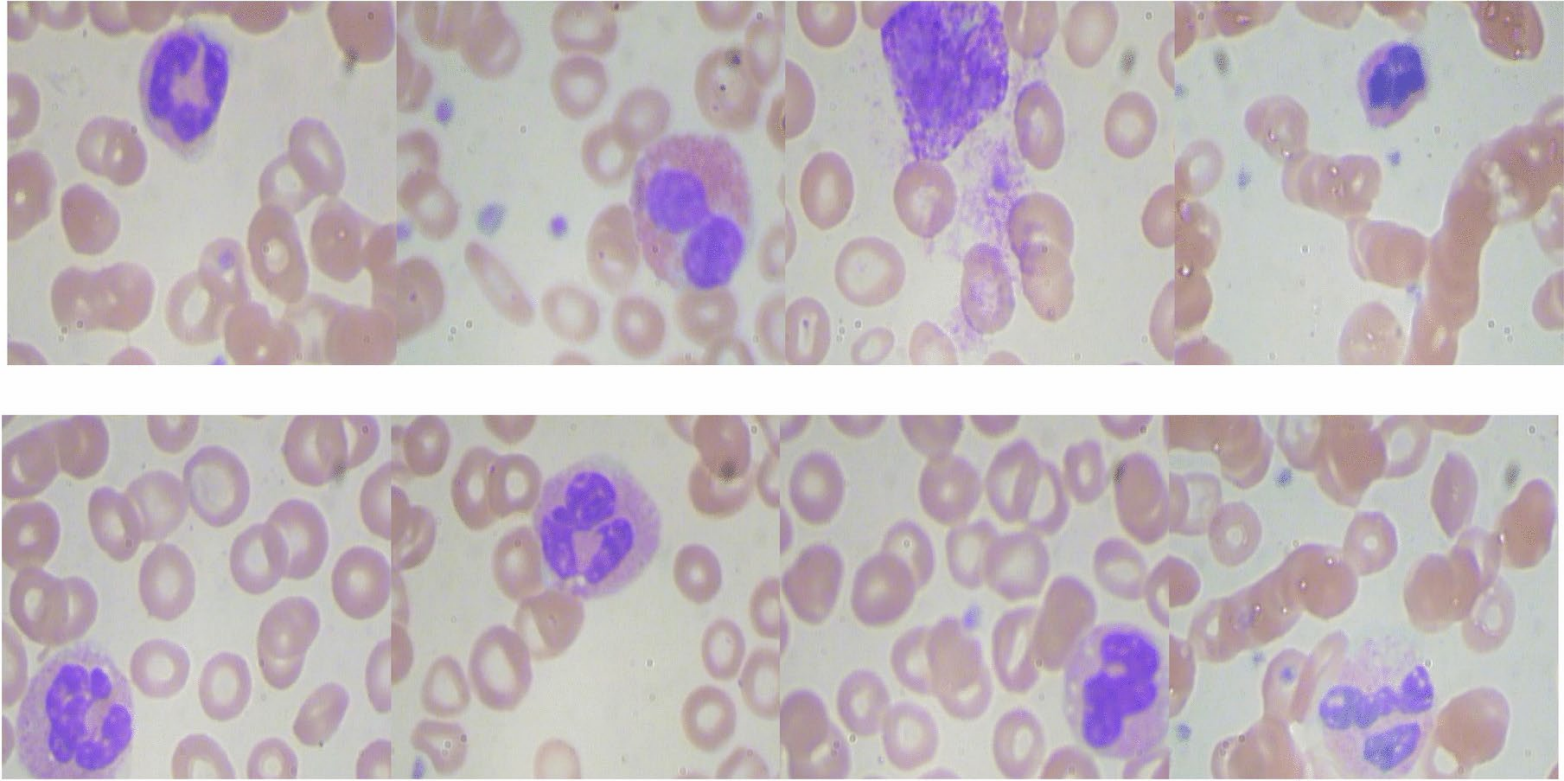
Shows a sample of the used dataset.

This article utilized a publicly available ALL dataset^33^. The mentioned dataset was used for training and evaluating the efficiency of the proposed algorithm. The dataset’s images were produced at Taleqani Hospital in Tehran, a facility specializing in bone marrow research. The dataset consisted of 3256 PBS images collected from 89 patients suspected of having ALL. PBS test is a method utilized by healthcare professionals to analyze red and white blood cells, as well as platelets. This test provides a clear image of changes in your blood cells and platelets that may be a sign of disease. The blood samples collected for staining were managed by skilled laboratory staff. Figure 9 shows a sample of the used dataset. The dataset is divided into two separate categories: benign and malignant. Hematogones are hematopoietic precursor cells that closely resemble cases of ALL. However, these cells are non-cancerous, resolve on their own, and do not require chemotherapy. ALL consists of three subtypes of malignant lymphoblasts: pro-B ALL, early pre-B ALL, and pre-B ALL. The images were taken with a Zeiss camera mounted on a 100× magnification microscope and stored as JPG files. A specialist used the flow cytometry instrument to determine the precise types and subtypes of these cells. Table 3 contains detailed information about this data set.

**Table 3:** The description of the used dataset.

Type	Subtype	Number of samples
Benign	Hematogones	504
Malignant	Early pre-B ALL	985
	Pre-B ALL	963
	Pro-B ALL	804
Total		3256

Actually, in the classification tasks, especially in medical domains such as leukemia classification, class imbalance occurs when one class (e.g., leukemia positive cases) is significantly underrepresented compared to another class (e.g., healthy cases). This can lead to a biased model that predicts the most common class more frequently. We can measure bias by using the following steps:

1. Class Bias: class bias can be determined by comparing the proportions of each class using the following equation:
  19 $$DR = \frac{X}{Y}$$
  where DR is the distribution ratio, $X$ is the number of cases in the majority class, and $Y$ is the number of cases in the minority class. In this case, if $DR>1$ this indicate an imbalance within the upper class, suggesting that the data set is skewed towards the majority class.
2. Class Weight (CW): in this method the weight of each class can be determined to account imbalance. In this paper, we have four classes; class1 (i.e., Hematogones), class 2 (i.e., Early pre-B ALL), class 3 (i.e., Pre-B ALL), and class 4 (i.e., Pro-B ALL) and the weight of each class can be determined as follows:
  20 $$CW_{n} = \frac{N}{{4*N_{n} }}$$
  where ${\text{CW}}_{\text{n}}$ is the class weight for $\text{n}$ class (n = 1, 2 3 4), $\text{N}$ is the total number of cases in the used dataset, and ${\text{N}}_{\text{n}}$ is the number of cases in $\text{n}$ class. Consequently, to deal with unbalanced class, assign higher weights to the minority class in the learning algorithm to make the model pay more attention to the minority class.

#### Example

As shown in Table 3, the total number of the used dataset is 3265 divided into benign (504 cases) which is class 1, and Malignant (2752 cases) that consists of three classes; class 2: Early pre-B ALL (985 cases), class 3: Pre-B ALL (963 cases), and class 4: Pro-B ALL (804 cases). To calculate the bias of each class:

*Step 1: Check the distribution of the classes*

- Total number of cases (N): N = 3256
- Number of cases in class 1: N_1_ = 504
- Number of cases in class 2: N_2_ = 985
- Number of cases in class 3: N_3_ = 963
- Number of cases in class 4: N_4_ = 804

*Step 2: Calculate DR between majority class and minority class*

- DR between class 1 (minority) and class 2 (majority)
  $$DR = \frac{985}{{504}} = 1.95$$
- DR between class 1(minority) and class 3 (majority)
  $$DR = \frac{963}{{504}} = 1.9$$
- DR between class 1 (minority) and class 4 (majority)
  $$DR = \frac{804}{{504}} = 1.59$$
- DR between class 3 (minority) and class 2 (majority)
  $$DR = \frac{985}{{963}} = 1.022$$
- DR between class 4 (minority) and class 2 (majority)
  $$DR = \frac{985}{{804}} = 1.22$$
- DR between class 4 (minority) and class 3 (majority)
  $$DR = \frac{963}{{804}} = 1.2$$

*Step 3: Interpretation*

The imbalance ratio clarifies the degree of the unequal distribution of classes. As shown in Step 2, according to DR, there is no significant variation between classes. The higher ratio between class 1 and class 4.

*Step 4: Actions to Address Class Imbalance*

In our case, there is no significant class imbalance. But, if there is high class imbalance, action should be taken to address this problem such as;

- Use data augmentation for minority classes such as Synthetic Minority Oversampling Technique** (**SMOTE).
- Use Class Weights: another way to deal with class imbalance is to use weight of classes. At first, calculate weight of each class using Eq. (20) then, use it in the training process for example if the class weight of the minority class in 2.5, this means that each case of this class will be treated with a weight of 2.5 during training.

### Evaluation metrics

Evaluation metrics including sensitivity, accuracy, error, and F-measure will be computed in the forthcoming tests. Table 4 shows the results of using a confusion matrix to find the values of various metrics. The confusion matrix can be summarized using a number of formulas, as given in Table 5.

**Table 4:** Confusion matrix.

		Predicted Class	
		1	0
Actual class	1	True Positive (TP)	False Negative (FN)
	0	False Positive (FP)	True Negative (TN)

**Table 5:** Confusion matrix formulas.

Measure	Formula	Definition
Precision (P)	$\frac{TP}{{\left( {TP + FP} \right)}}$	The accuracy rate of positive predictions
Specificity	$\frac{TN}{{\left( {TN + FP} \right)}}$	Specificity refers to the capacity to accurately identify individuals who do not have a particular disease as negative
Recall/Sensitivity (R)	$\frac{TP}{{\left( {TP + FN} \right)}}$	The proportion of correctly identified positive instances among all instances labed as positive
Accuracy(A)	$\frac{{\left( {TP + TN} \right) }}{{ \left( {TP + TN + FP + FN} \right)}}$	The accuracy ratepredictions
ror(E)	$1 - A$	The percentage of inaccurate predictions
F-measure	$\frac{2*PR}{{\left( {P + R} \right)}}$	Recall and precision are weighted harmonically averaged
Dice similarity coefficient (DSC)	$DSC = \frac{2TP}{{\left( {2TP + FP + FN} \right)}}$	DSC is a statistical measure used to quantify the similarity between two samples
Jaccard Index (JI)	$JI = \frac{{\left( {TL \cap PL} \right)}}{{\left( {TL \cup PL} \right)}}$	JI is a quantitative measure, ranging from 0 to 1 (equivalent to 0% to 100%), which assesses the similarity between two sets of samples

### Comparison of DAOA with standard meta-heuristics

**Fig. 10:**
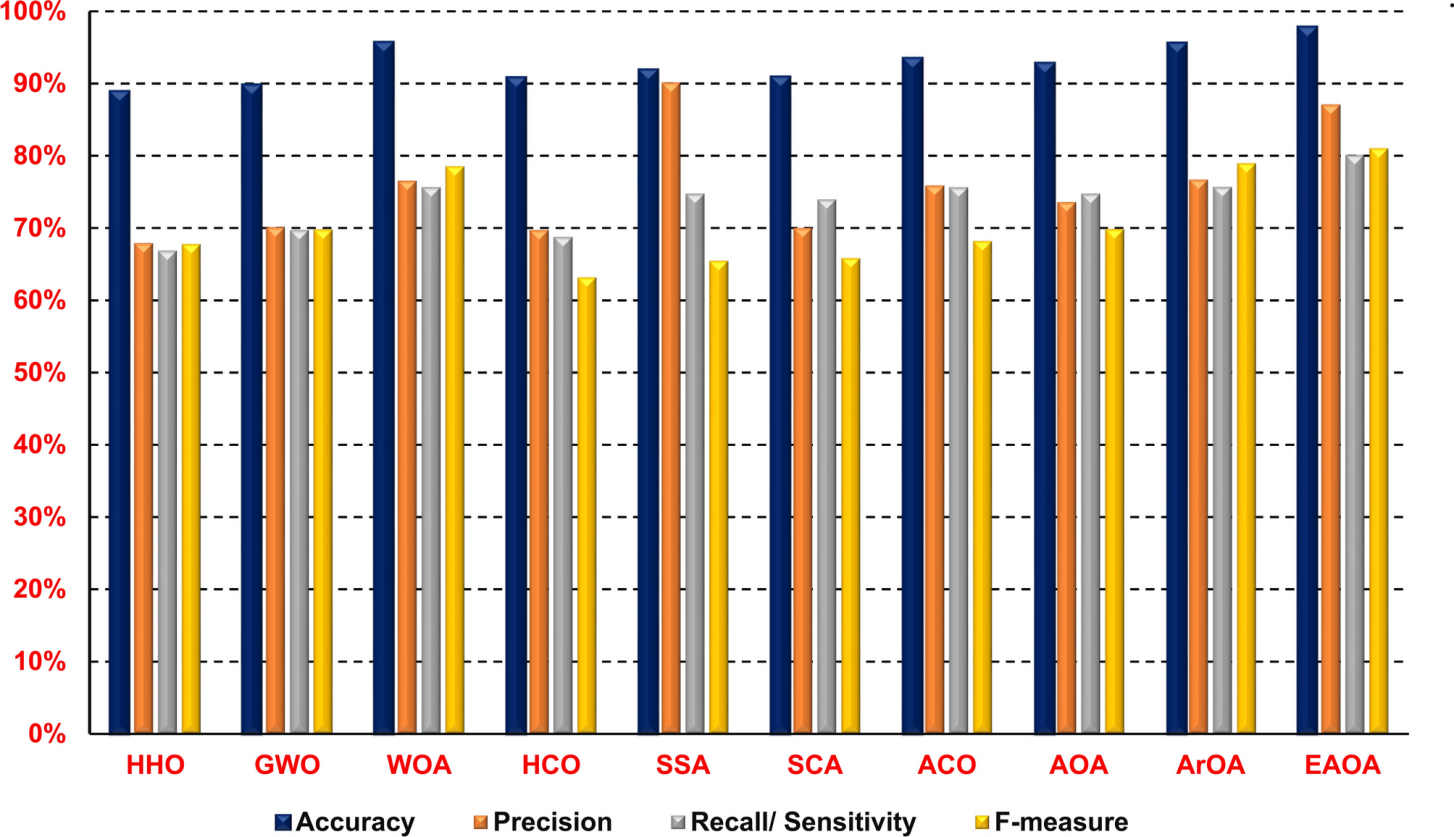
Comparison of DAOA with standard Meta-heuristics.

The Dimensional Archimedes Optimization Algorithm (DAOA) that has been proposed will be assessed here. A comparison of several feature selection techniques with the proposed DAOA feature selection methodology, which uses the NB classifier as the basis classifier, is made in order to argue the efficacy of the suggested method. The aforementioned techniques include Harris Hawks Optimization (HHO), Grey Wolf Optimizer (GWO), Whale Optimizer Algorithm (WOA), Salp Swarm Algorithm (SSA), Sine Cosine Algorithm (SCA), Ant Colony Optimization (ACO), Arithmetic Optimization Algorithm (AOA), and Archimedes Optimization Algorithm (ArOA). Figure 10 shows the outcomes.

The DAOA, as shown in Fig. 10, surpasses other meta-heuristic algorithms in accuracy, precision, recall/sensitivity, and f-measure. The model achieves an accuracy of 97.8%, precision of 87%, recall of 80.1%, and F-measure of 81.2%. DAOA relies on DLS, which enhances its performance.

### Testing the proposed leukemia classification system (LCS)

**Fig. 11:**
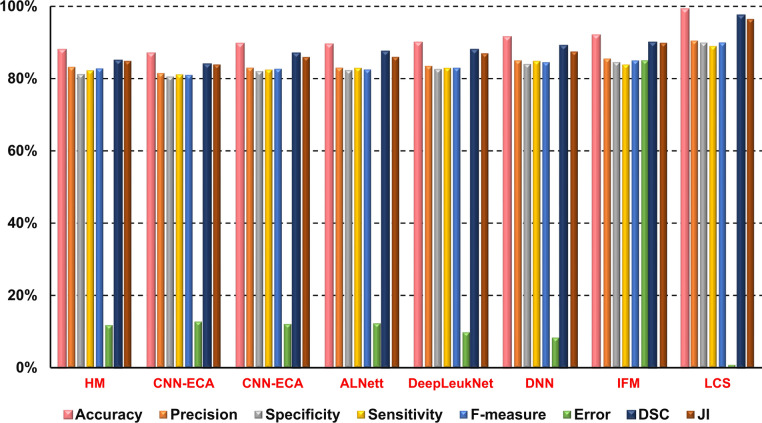
Comparison of the proposed LCS with different classification methods.

It is now time to assess the proposed LCS in this section. We compared our proposed system with several recently used methods for leukemia classification, as shown in Table 1, to evaluate its effectiveness. The methods listed are: HM^1^, CNN-ECA^17^, CNN^18^, ALNett^19^, DeepLeukNet^20^, DNN^21^, and IFM^22^. The proposed LCS utilizes all capabilities, employing DAOA for feature selection and EC for classification. The results can be found in Fig. 11.

Figure 11 demonstrates that the proposed LCS surpasses other leukemia classification methods in accuracy, precision, specificity, sensitivity, and F-measure. It introduces 99.2%, 90.5%, 89.9%, 89%, and 89.7% in that specific sequence. Moreover, it minimized errors to a value of 0.8%. It also offers a maximum DSC of 98% and JI of 96.5%. The proposed LCS relies on two crucial stages: FSS and CS. The most crucial features are selected through FSS using the highly effective method DAOA, which relies on DLS. The DLS mechanism transfers valuable information about the best position of the population in each generation to the personal best position of each particle. This improves both the precision and rapidity of convergence, while reducing the likelihood of the “two steps forward, one step back” scenario. This problem offers a more precise solution and also decreases the necessary time.

Then, these accurately selected features fed into the proposed classification model, which is based on EC. EC depends on the maximum voting for different classifiers. Actually, using EC refers to strategies that generate multiple models and subsequently merge them to yield enhanced outcomes. Ensemble methods in machine learning typically yield more precise solutions compared to individual models. Hence, the proposed LCS outperforms other competitors.

### Ablation studies: evaluating the impact of key components

Through this subsection, the importance of each component in the proposed model will be discussed to highlight their contribution in the proposed model. To achieve this aim, at first the proposed model will be evaluated without feature selection (Model 1). Then, testing the proposed model with traditional feature selection such as Particle Swarm Optimization (PSO) (Model 2). After that, testing the proposed model with extracting textural features only and select the most important features using DAOA (Model 3). Testing the proposed model with morphological features only and extract the most important features using DAOA (Model 4). Finally, testing the proposed model by using single classifier in Classification Stage (CS) such as RF, DT, and GB. Results are shown in Table 6.

**Table 6:** Evaluating the impact of key components of the proposed model.

Used technique	Accuracy (%)	Precision (%)	Specificity (%)	Sensitivity (%)	F-measure (%)
The proposed LCS	99.2	90.5	89.9	89	89.7
Model 1: with full features	96	83	82.5	81.9	82.45
Model 2: with PSO	96.4	83.8	80.9	80.9	82.32
Model 3: with texture features only	94	81.2	80	80	80.6
Model 4: with morphological features only	93.6	81.9	79.2	79.1	80.48
Model 5: with RF	96.5	83.6	81.3	81.2	82.38
Model 6: with DT	97	88.5	87.4	87	87.74
Model 7: with GB	96.1	82.9	81.7	82	82.57

As illustrated in Table 6, ablation studies were performed to identify components that significantly impact accuracy and reliability in leukemia classification. Each experiment isolates a key component of our proposed model, including feature extraction methods, feature selection, or classification strategies. The impact on model performance when that component is changed or removed is then evaluated. As presented in Table 6, when we use the full features (i.e., texture and morphological features) without performing feature selection process, the model introduces 96%, 83%, 82.5%, 81.9%, and 82.45% accuracy, precision, specificity, sensitivity, and F-measure at the same order. Moreover, when using texture features only, the model introduces an accuracy of 94%, while it introduces 93.6% accuracy when using morphological features only. Additionally, when single classifier used to perform classification rather than ensemble classifier, the model introduces 96.5% accuracy using RF, and 97% using DT, and 96.1% using GB. Finally, the results obtained illustrate that the proposed model introduces the maximum accuracy, precision, specificity, sensitivity, and F-measure. the reason is that, the proposed LCS depends on the most important features which are selected using a very effective wrapper method (i.e. DAOA), and ensemble classifier that introduces the final decision based on a maximum voting.

### Time complexity analysis

The time complexity of an algorithm is a crucial determinant in evaluating its performance. Time complexity measures the duration required for its execution as a function of the input size. Consequently, time complexity of each phase are illustrated as follows:

i. Time complexity for Image Processing stage (IPS)
  Resizing: assume that $H$ and $W$ are the height and width of the original image and we will resizing it to 500*500 the time complexity is $O\left(H*W\right).$
  Image enhancement: time complexity for enhancing image depend on the number of pixels in the image. Consequently, the time complexity will be $O\left(H*W\right).$
  Filtering: the complexity of applying the Gaussian filter of kernel size $k*k$ is $O(H\times W\times {k}^{2})$

Finally, for small $k$, the final time complexity for IPS is $O\left(H\times W\right).$

ii. Time complexity for image segmentation Stage (ISS)

The overall time complexity for ISS when K-means and Watershed algorithms are run sequentially is $O(N\times K)$, where $N$ is the number of pixel in the image and $K$ is the number of clusters for K-means.

iii. Time complexity for Feature Selection Stage (FSS)

According to AOA analysis, the time complexity of conventional AOA is $O(N+C)$. Thus, the time complexity of DAOA is $O(T(D+N)+(C\times N))$, where T denotes the number of iterations, $D$ signifies the problem’s dimension, $N$ indicates the population size, and $C$ represents the cost of the objective function.

iv. Time complexity for Feature Selection Stage (FSS)

The total time complexity of RF, DT, GB, and AdaBoost where they combining together and the final decision is based on the maximum voting is:

- For training: $$O(T\times N\times F\times log(N))$$
- For testing: $$O(T\times log(N)+M)$$

where $T$ is the number of trees in each classifier, $N$ is the number of training samples, $F$ is the number of features, and $M$ is the number of test samples.

## Discussion

In this paper, a new model based on AI is introduced to detect and classify leukemia patient. The proposed system is LCS that is based on PBS images. The proposed LCS is mainly based on the informative feature that are selected using DAOA. DAOA achieved accuracy of 97.8%, precision of 87%, recall of 80.1%, and F-measure of 81.2% outperforms the other competitors. Additionally, the classification results indicate that, despite the simplicity of our proposed method, it achieves satisfactory performance for leukemia diagnosis. Therefore, the proposed algorithm may serve as a supplementary diagnostic instrument for pathologists. It outperforms alternative leukemia classification methods in accuracy, precision, specificity, sensitivity, and F-measure. It presents 99.2%, 90.5%, 89.9%, 89%, and 90% respectively. The proposed LCS demonstrates superior adaptability, robustness, and classification accuracy compared to contemporary leukemia classification methodologies.

## Conclusions and future works

This paper introduces a novel system to classify leukemia patients based on AI which is called Leukemia Classification System (LCS). The proposed system consists of Image Processing Stage, Image Segmentation Stage, Feature Extraction Stage, Feature Selection Stage, and Classification Stage. In LCS, two contributions are introduced. The first is new feature selection methodology, which is Dimensional Archimedes Optimization Algorithm (DAOA). Actually, DAOA combines between Dimensional Learning Strategy (DLS) and Archimedes Optimization Algorithm (AOA). Actually, DLS helps AOA to provide an efficient and optimal solution. The second contribution is the proposed classification model which is Ensemble Classifier (EC) to classify leukemia patients. EC is based on the maximum voting of several classifiers, which are; Random Forest (RF), Decision Tree (DT), Gradient Boosting (GB), and Adaptive Boosting (AdaBoost). The experimental results show that the proposed LCS performs better than other competitors in terms of accuracy, precision, specificity, recall, F-measure, DSC, and JI. It introduces about 99.2%, 90.5%, 89.9%, 89%, 90%, 98%, and 96.6%, respectively.

The limitations of the proposed LCS model are that it relies on the availability of high-quality labeled data for training purposes. The effectiveness of the model may be reduced, especially in the context of infrequent blood cell types when the dataset is constrained. Imbalanced datasets pose a significant challenge in medical image analysis, especially in blood cell classification. Future research may focus on developing methodologies to address imbalanced datasets, including generative adversarial networks (GANs) and various data augmentation strategies. The predictive performance and diagnostic accuracy of the model can be improved by incorporating data from additional media, including blood cell morphology, immunophenotyping data, and clinical metadata. Future research may investigate techniques to effectively fuse data from multiple media to enhance the accuracy of blood cell classification and provide more comprehensive diagnostic insights. Incorporating supplementary information, such as patient history, may enhance the overall accuracy.

## Data Availability

The used dataset is available at https://www.kaggle.com/paultimothymooney/blood-cells/version/6.
